# LC-MS/MS-Based Metabolomics Identifies 2-Aminopurine as a Predictive Freshness Biomarker in Goose Egg Yolk During Refrigerated Storage

**DOI:** 10.3390/foods15030588

**Published:** 2026-02-06

**Authors:** Suyu Fan, Laidi Wang, Yuchun Cai, Hongyan Sun, Wenming Zhao, Guohong Chen, Youqing Bian, Yang Zhang

**Affiliations:** 1 Jiangsu Key Laboratory for Animal Genetic, Breeding and Molecular Design, College of Animal Science and Technology, Yangzhou University, Yangzhou 225009, China; fansuyu20030729@163.com (S.F.); yzwanglaidi@163.com (L.W.); 211902102@stu.yzu.edu.cn (Y.C.); sunhy@yzu.edu.cn (H.S.); wmzhao@yzu.edu.cn (W.Z.); ghchen@yzu.edu.cn (G.C.); 2 Institute of Epigenetics and Epigenomics, College of Animal Science and Technology, Yangzhou University, Yangzhou 225009, China; 3 Jiangsu Agri-Animal Husbandry Vocational College, Taizhou 225300, China

**Keywords:** goose egg yolk, metabolomics, 2-aminopurine, purine metabolism, freshness biomarker

## Abstract

Goose yolk, the primary source of nutrients and flavor, is particularly susceptible to quality deterioration during storage, yet its metabolic dynamics remain poorly characterized. To elucidate these changes, we combined physicochemical assays with untargeted LC-MS/MS metabolomics to systematically profile the temporal metabolic alterations in goose egg yolks stored at 4 °C for up to 60 days, using day-1 yolks as fresh controls. Our analysis quantified 1005 metabolites and identified a critical metabolic shift occurring after 30 days of storage. Among 21 significantly altered metabolites, the sustained decline of adenosine and 2-aminopurine, alongside the accumulation of 4-hydroxyretinoic acid, strongly correlated with the loss of egg freshness. Interaction network and pathway analyses pinpointed purine metabolism—with adenosine and 2-aminopurine as central nodes—as a core pathway impaired in yolk during storage. Crucially, we identify 2-aminopurine as a novel, storage-sensitive biomarker for goose egg freshness derived from yolk metabolomics, directly linking intracellular metabolic dysregulation to observable quality decline. This study deciphers the metabolic landscape of goose egg yolk aging and provides a targeted, mechanism-based strategy for yolk-centric quality monitoring and preservation, offering new insights for food composition analysis and safety assurance.

## 1. Introduction

Eggs are a nutrient-dense food source of high-quality protein, vitamin B12, and essential fatty acids, and they contain several other bioactive compounds for human health [1]. Compared to chicken eggs, goose eggs are prized for their rich flavor, larger yolk-to-albumen ratio, and distinct nutritional profile; thus, they occupy a unique niche in global cuisines, particularly in Asia and Europe [2]. Presently, this practice poses substantial challenges to both food safety and human health. This is primarily due to the susceptibility of goose eggs to quality deterioration during storage, which is further compounded by the biochemical complexity inherent to goose eggs. The quality and safety of goose eggs is determined by external and internal egg quality traits, which are profoundly influenced by storage conditions. Generally, external egg quality traits include egg weight, shell color, shell thickness, shape index and breaking strength [3,4]. Internal egg quality parameters mainly include yolk index, albumen index, yolk color and Haugh unit. Egg yolk and albumen are two important nutrients for human consumption [5]. Accumulated evidence has recently highlighted that preservation period and conditions directly affect eggs’ quality of albumen and egg yolk from chicken [6,7,8], duck [5,9,10] and goose [2]. Currently, this evidence has been widely used to extend the shelf life of eggs using traditional methods of egg preservation, such as refrigeration and coating. However, the specific metabolic changes occurring in goose eggs under different preservation periods and conditions, including the key metabolic factors involved in egg quality changes, have not been fully elucidated.

Metabolomics has emerged as a powerful platform for identifying and quantifying a wide range of metabolites [11], providing unprecedented insights into metabolic pathway dynamics during food storage. Several studies offer a more holistic view of the changes in egg quality over time. Recent studies using metabolomics have revealed significant changes in the metabolite composition of eggs during long-term storage [9]. It was found that metabolite composition changes in the albumen and yolk of fresh duck eggs stored for 3 weeks [9]. Unlike conventional quality assessment methods, metabolomics can identify early-stage biochemical changes in biochemical processes that affect egg quality and safety, offering predictive capabilities for shelf-life estimation. It was hypothesized that analogous metabolic alterations would be found in the egg yolk of goose eggs during extended storage periods. However, extant research in this domain remains sparse.

Liquid chromatography–tandem mass spectrometry (LC–MS/MS) is a central analytical platform in metabolomics, providing the sensitivity, specificity and dynamic range needed to profile complex matrices such as avian eggs [12,13]. Its combination of separation and accurate mass detection makes it a gold-standard tool for comprehensive metabolite analysis. However, access is often limited by high costs, specialized expertise and demanding sample preparation, restricting its use in resource-constrained laboratories. For studies involving widely distributed biological materials—such as goose eggs, which vary across regions and continents—institutional and cross-regional collaboration therefore becomes essential. Sharing instrumentation, knowledge and protocols not only make such research feasible but also improves data comparability and methodological rigor. This cooperative approach represents a practical strategy for advancing agricultural and food-science research when individual lab resources are insufficient.

Here, we investigate the impact of preservation period on the egg yolk quality of goose eggs during prolonged refrigeration using liquid chromatography–mass spectrometry/mass spectrometry (LC-MS/MS). Metabolomic profiling of goose eggs revealed stage-specific remodeling, with a critical freshness transition occurring between 30–60 days. Crucially, network analysis established 2-Aminopurine, a key hub in purine metabolism, as a promising novel biomarker for predicting goose egg freshness. The present study reveals the metabolic adaptations in goose eggs during long-term storage and provides valuable insights for developing more effective preservation strategies.

## 2. Materials and Methods

### 2.1. Experiment Design and Sample Collection

**Figure 1:**
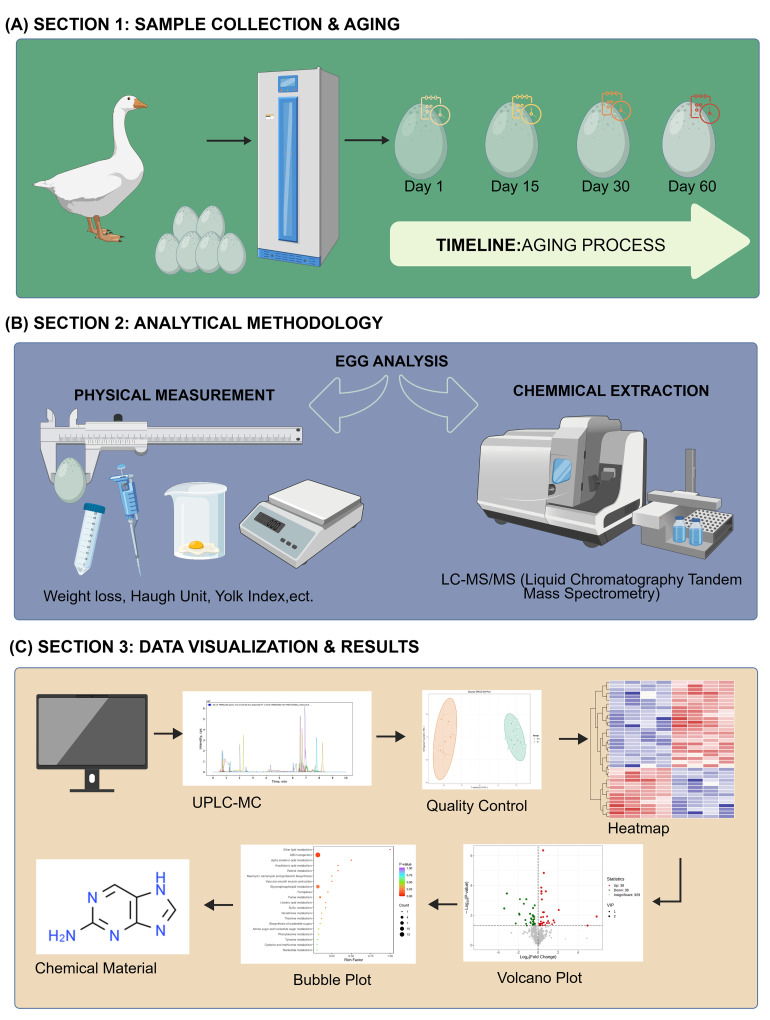
**Schematic overview of the experimental design and comprehensive analytical workflow for Taihu goose eggs.** (**A**) Schematic diagram illustrating the sample collection and aging process, where Taihu goose eggs were collected and stored at 4 °C for 1, 15, 30, and 60 days to simulate different shelf-life stages. (**B**) Schematic summary of the dual analytical methodology used in the study. The workflow divides into physical quality measurements (determining weight loss, Haugh unit, and yolk index) and chemical extraction followed by LC-MS/MS analysis for non-targeted metabolomics profiling. (**C**) Comprehensive data processing and bioinformatics visualization pipeline. The sequence depicts the transition from raw UPLC-MS data acquisition and Quality Control (QC) assessment to multivariate statistical analyses—including Heatmaps, Volcano plots, and Bubble plots—ultimately leading to the identification of key chemical biomarkers.

Taihu goose eggs were provided by Yangzhou University. All eggs were collected on the same day from a healthy flock within the same farm batch to ensure uniformity in initial physiological state and to minimize batch effects. A total of 24 Taihu goose eggs with similar physiological states were selected and stored at 4 °C for 1, 15, 30 and 60 days, respectively. Goose egg yolks stored for 1 day were classified as fresh controls, while those stored for extended periods (15, 30, and 60 days) were designated as long-term storage groups. Egg yolk samples were collected at each storage period and then carefully transferred to sterile syringes for subsequent metabolomic analysis. A schematic overview of the complete experimental design and analytical workflow is provided in Figure 1. The sample size (*n* = 6/time point) aligns with comparable metabolomics studies, and a post hoc sensitivity analysis confirmed robust detection of medium-to-large effects. The yolk-focused analysis targeted the relevant lipid and antioxidant pathways, while Haugh unit measurements were integrated to acknowledge albumen interactions.

### 2.2. Sample Preparation and Extraction

Yolk samples were thawed on ice, and 20 mg (±1 mg) of each sample was weighed into a centrifuge tube. A 400 μL aliquot of extraction solution (70% methanol in water containing internal standards) was added, followed by vortexing for 3 min. Samples that did not disperse well were vortexed for an additional 3 min with steel beads. The mixture was sonicated in an ice–water bath for 10 min, vortexed for 1 min, and incubated at −20 °C for 30 min to precipitate proteins. After centrifugation at 12,000 r/min for 10 min at 4 °C, 300 μL of the supernatant was transferred to a new tube. A second centrifugation was performed at 12,000 r/min for 3 min at 4 °C, and 200 μL of the final supernatant was transferred to a vial insert for LC-MS/MS analysis.

### 2.3. Ultra Performance Liquid Chromatography–Tandem Mass Spectrometry (UPLC-MS/MS) Conditions

Chromatographic Separation. Separation was performed using an ExionLC™ AD system (Sciex, Framingham, MA, USA) equipped with a Waters ACQUITY UPLC HSS T3 C18 column (1.8 µm, 2.1 mm × 100 mm). The mobile phase consisted of solvent A (0.1% formic acid in water) and solvent B (0.1% formic acid in acetonitrile). The gradient elution program was set as follows: 0 min, 5% B; 2.0 min, 20% B; 5.0 min, 60% B; 6.0 min, 99% B; 7.5 min, 99% B; 7.6 min, 5% B; 10.0 min, 5% B. The flow rate was 0.4 mL/min, the column temperature was maintained at 40 °C, and the injection volume was 2 µL.

Mass Spectrometry Data Acquisition. MS/MS analyses were performed using a QTRAP^®^ LC-MS/MS System (Sciex) equipped with an ESI Turbo Ion-Spray interface, operating in both positive and negative ion modes and controlled by Analyst 1.6.3 software (Sciex). The ESI source parameters were optimized as follows: source temperature 500 °C; ion spray voltage (IS) 5500 V (positive mode) and −4500 V (negative mode); ion source gas I (GSI), gas II (GSII), and curtain gas (CUR) set at 55, 60, and 25 psi, respectively; and collision-activated dissociation (CAD) set to high. Instrument tuning and mass calibration were performed with 10 and 100 μmol/L of polypropylene glycol solutions in QQQ and LIT modes, respectively.

### 2.4. Metabolite Identification and Quantification

Metabolite identification was conducted based on the self-built Metware Database (MWDB) using retention time (RT), precursor/product ion pairs, and MS/MS fragmentation data. Quantification was performed in multiple reaction monitoring (MRM) mode using triple quadrupole mass spectrometry. In this mode, quadrupole Q1 selected the precursor ion to eliminate interference, and Q3 filtered the specific fragment ion derived from collision-induced dissociation in Q2, ensuring high specificity and sensitivity. Specific MRM transitions were monitored for each metabolite based on the curated information in the MWDB. Chromatographic peak areas were integrated and aligned using MultiQuant 3.0.3 software (Sciex).

### 2.5. Data Preprocessing and Statistical Analysis

Data Preprocessing. To ensure data integrity, missing values were imputed with one-fifth of the minimum positive abundance recorded for the corresponding metabolite. The stability of the analytical system was assessed using quality control (QC) samples. The coefficient of variation (CV) was calculated for each metabolite across QC samples, and metabolites with CV > 30% were excluded from the final dataset to retain only reproducible features; this step resulted in the removal of 134 metabolites (11.8% of the total 1139), leaving 1005 highly reproducible features for subsequent analysis. The resulting data were normalized by unit variance (UV) scaling prior to statistical analysis.

Statistical Analysis. Statistical processing was performed using R software (version 4.4.2).

Multivariate Analysis: Unsupervised Principal Component Analysis (PCA) and t-Distributed Stochastic Neighbor Embedding (t-SNE) were employed to assess the intrinsic variation and clustering trends within the dataset. Subsequently, Orthogonal Partial Least Squares Discriminant Analysis (OPLS-DA) was conducted to maximize group separation and identify discriminating metabolites. The reliability of the OPLS-DA model was evaluated using permutation tests (n = 200) and validated by R^2^Y and Q^2^ metrics.

Differential Metabolite Screening: Significantly differentially accumulated metabolites (DAMs) were identified using a dual-criterion strategy combining multivariate and univariate analysis. Specifically, metabolites were selected if they exhibited a Variable Importance in Projection (VIP) score ≥ 1.0 (extracted from the OPLS-DA model) and a statistical significance of *p* < 0.05 (determined by a two-tailed Student’s *t*-test).

Clustering and Correlation: Hierarchical cluster analysis (HCA) was performed using the Complex Heatmap R package on UV-scaled data. Pearson correlation analysis was conducted to explore relationships between metabolites and egg quality traits.

### 2.6. Pathway Enrichment Analysis

Identified metabolites were annotated using the KEGG Compound database and mapped to the KEGG Pathway database. Metabolite Set Enrichment Analysis (MSEA) was performed to identify biologically meaningful patterns using MetaboAnalystR [11,12]. To quantify the core pathways associated with yolk quality deterioration, we identified significantly enriched metabolic pathways (*p* < 0.05). The results were visualized using bubble plots generated with the ggplot2 package in R.

### 2.7. Statistical Analysis for Quality Traits

For basic egg quality traits (e.g., Haugh unit, yolk index) and targeted verification data, statistical analysis was performed using GraphPad Prism 10 (GraphPad Software, San Diego, CA, USA). Data are presented as means ± SEM. Differences between groups were assessed using a two-tailed unpaired Student’s *t*-test or one-way ANOVA where appropriate. Significance levels were defined as * *p* < 0.05 and ** *p* < 0.01.

## 3. Results

### 3.1. Basic Goose Egg Quality Parameters During Refrigeration

**Figure 2:**
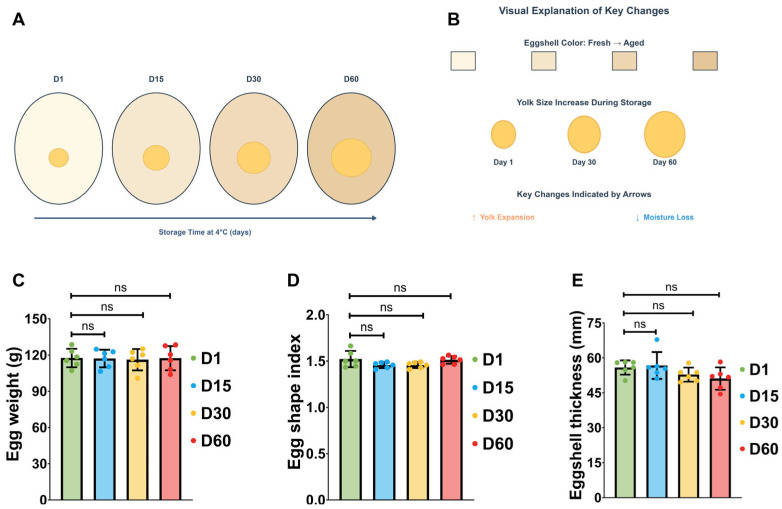
**Effects of storage duration on Taihu goose egg quality parameters.** (**A**) Schematic diagram illustrating Taihu goose eggs stored at 4 °C for 1, 15, 30, and 60 days, depicting the visual changes in eggshell color and yolk size over time. **(B)** Schematic summary of key quality alterations during storage, including the transition in eggshell color and the increase in yolk size. (**C**) Changes in final egg weight across storage durations (1, 15, 30, 60 days). (**D**) Changes in shell index across storage durations (1, 15, 30, 60 days). (**E**) Changes in eggshell thickness across storage durations (1, 15, 30, 60 days). Data represent mean ± SD, *n* = 6. ns *p* > 0.05 (two-tailed Student’s *t*-test).

The random selection of Taihu goose eggs resulted in initial egg weight variation based on the storage duration (1, 15, 30, and 60 days, see Figure 2A). The results indicated that the final weight of the goose eggs after prolonged storage varied according to the storage duration (see Figure 2B). However, after covariate analysis, there were no differences in the final egg weight and all egg quality parameters (such as the ratio of shell shape index and shell thickness) among the storage times. Compared to the eggs stored for 1 day, those stored for 60 days exhibited the highest shell shape index (see Figure 2C). There were no differences in shell weights among the various treatments post-drying. In comparison to the eggs stored for 1 day, the eggs stored for 15, 30, and 60 days exhibited lower shell thickness. A notable observation was that the eggs stored for 30 and 60 days exhibited the lowest shell thickness (see Figure 2D). In terms of increasing storage duration for breeding eggs, these parameters do not appear to exhibit any particular trend.

### 3.2. Progressive Nutrient Redistribution in Goose Eggs During Storage

**Figure 3:**
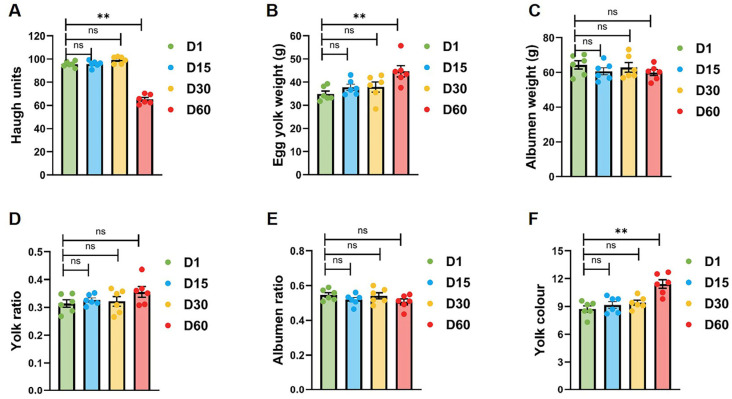
**Temporal degradation of nutritional and structural parameters in goose eggs during extended storage.** Changes in Haugh units (**A**), albumen weight (**B**), yolk weight (**C**), yolk ratio (**D**), albumen ratio (**E**) and yolk color (**F**) across storage durations (1, 15, 30, 60 days). Data represent mean ± SEM, *n* = 6. ns *p* > 0.05 and ** *p* < 0.01 (two-tailed Student’s *t*-test).

To further investigate temporal changes in the nutritional architecture of stored goose eggs, we analyzed key quality parameters, including Haugh units (egg white integrity), yolk color (pigmentation stability), albumen ratio (protein content distribution), and yolk ratio (lipid compartmentalization). The Haugh unit, a key indicator of egg white quality, maintained relative stability during the first 30 days before precipitously decreasing by 31.7 ± 2.5% between days 30–60 (Figure 3A). During the storage period, the weight of the egg white decreased linearly from Day 1 to Day 60, while the weight of the yolk increased linearly (Figure 3B,C). The ratio of egg white to yolk also exhibited corresponding changes, with the egg white ratio decreasing from 0.55 ± 0.01 (Day 1) to 0.51 ± 0.02 (Day 60) (*p* = 0.004), and the yolk ratio increasing from 0.31 ± 0.01 (Day 1) to 0.36 ± 0.02 (Day 60) (*p* = 0.004), indicating moisture migration (Figure 2D,E). The yolk pigmentation exhibited significant degradation during storage, with Roche color fan scores decreasing linearly from 9.7 ± 0.7 to 9.1 ± 0.7 (6.7% reduction) over the 60-day period (Figure 3F). The majority of parameters attained inflection points within a time frame of 15–30 days, indicating that this interval signifies a critical threshold for the maintenance of quality. A comprehensive analysis revealed a progressive deterioration of goose egg quality with increasing storage duration.

### 3.3. Metabolic Profiling of Goose Eggs During Refrigeration

**Figure 4:**
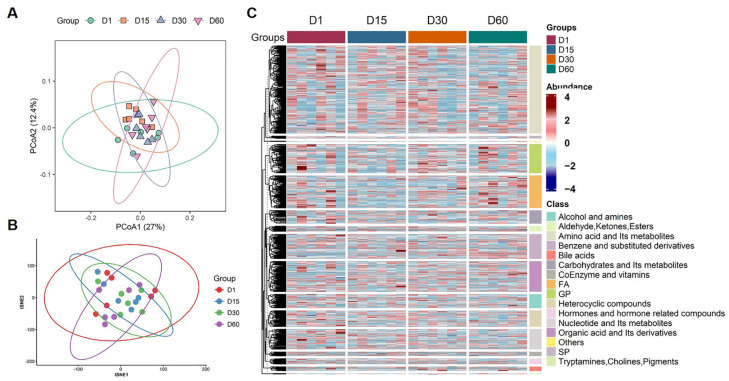
**Metabolic profiling of Taihu goose eggs during refrigeration.** (**A**) Principal component analysis (PCA) distributed individual time-specific samples across storage durations (1, 15, 30, 60 days). (**B**) The non-linear T-distributed stochastic neighbor embedding (t-SNE) dimensionality reduction algorithms clustered individual time-specific samples across storage durations (1, 15, 30, 60 days). (**C**) Heatmap of 1005 metabolites across 16 super classes in 24 samples across storage durations (1, 15, 30, 60 days).

To investigate the metabolic adaptations in goose eggs during prolonged refrigeration, we analyzed the yolk of Taihu goose eggs stored for 1, 15, 30 and 60 days. In total, untargeted metabolomics analysis with liquid chromatography–mass spectrometry (LC-MS/MS) was performed on 24 samples (Figure 4A). After peak alignment, quality control and normalization, a total of 1005 metabolites across 16 super classes were identified in all samples, with classification accuracy validated against HMDB (57.7% match rate) databases (Supplementary File S1).

A total of 588 compounds (58.5%) were successfully matched to known metabolic pathways in the KEGG database. Subsequently, the metabolic data was subjected to principal component analysis (PCA) and T-distributed stochastic neighbor embedding (t-SNE), and the analysis result revealed that the samples were distributed according to their different storage durations (Figure 4A,B). Heatmap analysis further indicated four distinct metabolic phases with characteristic molecular signatures during different storage durations (Figure 4C). These findings indicate that Taihu goose eggs yolks undergo significant temporal metabolic remodeling during refrigeration.

### 3.4. Global Metabolic Shifts in Goose Eggs During Refrigeration

**Figure 5:**
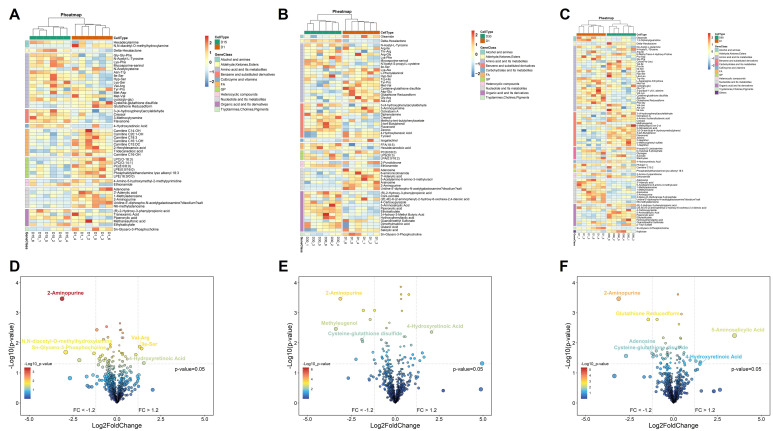
**Global metabolic shifts in Taihu goose eggs during refrigeration.** Heatmap analysis shows the distribution of significantly altered metabolites detected in the yolk of Taihu goose eggs on days 15 (**A**), 30 (**B**) and 60 (**C**). Volcano plot shows the distribution of significantly altered metabolites detected in the yolk of Taihu goose eggs on days 15 (**D**), 30 (**E**) and 60 (**F**).

Untargeted metabolomics analysis of goose eggs revealed profound temporal changes in metabolic composition when comparing each storage duration (Day 15, 30 and 60) to fresh eggs (Day 1 controls). Using stringent criteria (VIP ≥ 1 and *p* < 0.05), we identified 52, 61, and 76 significantly altered metabolites after 15 days, 30 days, and 60 days of storage, respectively (Figure 5A–C). After 15 days of storage, the top significantly altered metabolites included 2-Aminopurine, N, N-diacetyl-O-methyl hydroxylamine, Sn-Glycero-3-Phosphocholine, Val-Arg, Ile-Ser, and 4-Hydroxyretinoic Acid (Figure 5D). After 30 days of storage, significant metabolic shifts indicative of oxidative stress began to emerge. Key altered metabolites included 2-Aminopurine, Methyleugenol, Cysteine-glutathione disulfide, and 4-Hydroxyretinoic Acid (Figure 5E). Notably, the accumulation of Cysteine-glutathione disulfide suggests an alteration in the redox balance within the yolk. After 60 days of storage, this trend intensified. The top significantly altered metabolites included 2-Aminopurine, Glutathione Reduced form (downregulated), Adenosine, Cysteine-glutathione disulfide (upregulated), 5-Aminosalicylic Acid, and 4-Hydroxyretinoic Acid (Figure 5F). The depletion of reduced Glutathione alongside the accumulation of its oxidized derivatives provides molecular evidence of progressive oxidative deterioration in the yolk during prolonged refrigeration, corroborating the decline in physical quality traits.

In summary, a notable finding was that 4-hydroxyretinoic acid (an oxidation-related retinoid) and 5-aminosalicylic acid exhibited significant upregulation across storage durations compared to fresh eggs. In contrast, 2-aminopurine and UDP-GalNAc-2Na demonstrated consistent downregulation over the same timeframe.

### 3.5. Prolonged Storage Remodels Nucleotide Metabolic Hubs

**Figure 6:**
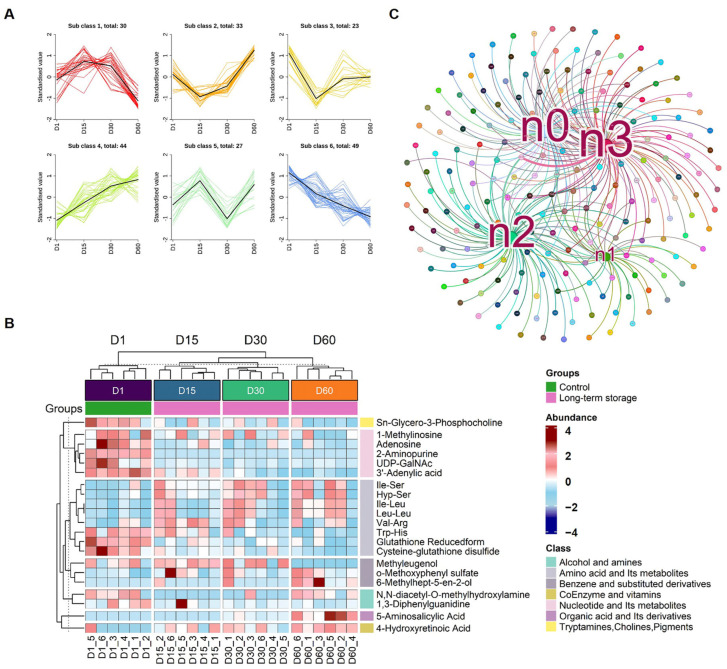
**Persistent nucleotide-centric metabolic suppression in refrigerated goose eggs.** (**A**) Composition of altered metabolites reveals persistent downregulation as the dominant category during refrigeration. (**B**) Heatmaps of highly dysregulated metabolites during refrigeration (Day 15, 30, 60 vs. Day 1). (**C**) Interaction network of dysregulated metabolites delineates a cohesive architecture centered on hub metabolites n0, n2 and n3.

It is noteworthy that the category of persistently downregulated metabolites constituted the most abundant group of altered compounds in refrigerated goose eggs (Figure 6A). Hierarchical clustering analysis of significantly dysregulated metabolites (|log_2_FC| ≥ 2) further revealed pronounced alterations concentrated specifically within the Nucleotide and Its Metabolites subclass (Figure 6B). Key representatives included Adenosine and 2-Aminopurine, both markedly suppressed during refrigeration. Interaction network analysis of these highly dysregulated metabolites (|log_2_FC| ≥ 2) delineated a cohesive metabolic network architecture (Figure 6C). This network centered on two hub metabolites: Adenosine (n3) and 2-Aminopurine (n0), which were identified as critical factors in cold storage-induced metabolic reprogramming.

### 3.6. Prolonged Storage Remodels 2-Aminopurine-Centric Purine Metabolism

**Figure 7:**
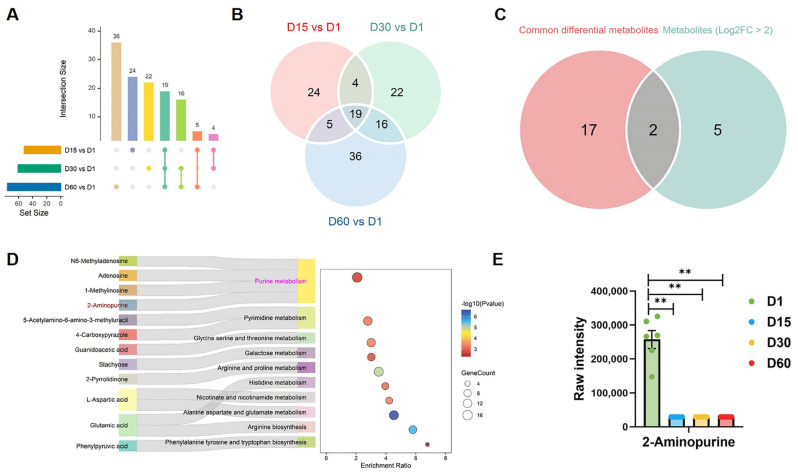
**Prolonged refrigeration remodels purine metabolism centered on 2-Aminopurine in goose egg yolks.** (**A**,**B**) Venn diagrams identify 19 common differentially altered metabolites (*p* < 0.05) during refrigeration (Day 15, 30, 60 vs. Day 1). (**C**) Venn diagrams of 19 common and 7 highly dysregulated metabolites (|log_2_FC| ≥ 2 and *p* < 0.05). (**D**) Metabolite set enrichment analysis of all metabolites reveals the top 10 significantly enriched pathways (*p* < 0.05) during refrigeration (Day 15, 30, 60 vs. Day 1). (**E**) Expression of 2-Aminopurine in goose egg yolks during refrigeration (Day 15, 30, 60 vs. Day 1). ** *p* < 0.01.

To further investigate the core and common metabolic alterations during goose egg refrigeration, we performed pairwise comparisons among samples (Day 15/30/60 vs. Day 1) to look for overlap metabolites. Pairwise comparisons across refrigeration time points revealed 19 common differentially altered metabolites during goose egg refrigeration (Figure 7A,B). The intersection of 19 common differentially altered metabolites (*p* < 0.05) and highly dysregulated metabolites (|log_2_FC| ≥ 2 and *p* < 0.05) further revealed two key metabolites: 2-Aminopurine and UDP-GalNAc-2Na (Figure 7C). Next, we conducted metabolite sets enrichment analysis (MSEA) using the reference metabolic pathway databases HMDB and KEGG on all metabolites to identity the core metabolites and key pathways involved in yolk spoilage. MSEA results revealed the top 10 significantly enriched metabolic pathways (*p* < 0.05) in goose eggs stored at 4 °C compared to fresh controls (Figure 7D). Among these pathways, purine metabolism was the most significantly perturbed. Within this pathway, 2-Aminopurine exhibited the most pronounced depletion during refrigeration (Figure 7D,E). These results suggest that 2-Aminopurine-centric purine metabolism emerges as a mechanistic bridge between refrigeration stress and yolk corruption.

### 3.7. 2-Aminopurine Is Associated with Goose Egg Quality Traits and Metabolic Shifts

**Figure 8:**
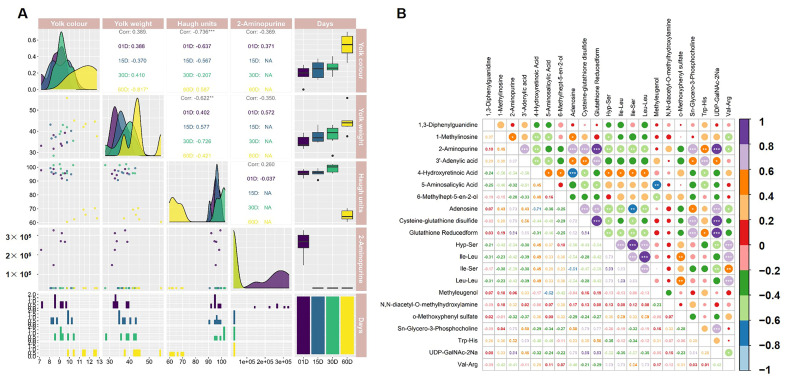
Correlation analysis revealed that 2-Aminopurine is associated with goose egg quality traits and metabolic shifts during storage. (**A**) Correlation analysis of 2-Aminopurine and key quality traits (yolk quality, egg weight, and Haugh units). (**B**) Correlation heatmaps of 2-Aminopurine and 20 highly dysregulated metabolites. The significant differences from low to high were *, **, and *** respectively.

The present study was conducted with the objective of investigating the role of 2-Aminopurine in goose egg preservation. To this end, an analysis was performed of its correlation with key quality traits (yolk quality, egg weight, and Haugh units) alongside highly dysregulated metabolites. Pearson correlation analysis demonstrated that 2-Aminopurine positively correlated with Haugh units but negatively correlated with yolk quality and egg weight (Figure 8A). Haugh Units themselves were negatively correlated with yolk quality and egg weight (Figure 8A). Furthermore, 2-Aminopurine exhibited significant positive correlations with metabolites associated with antioxidant capacity, structural integrity, and energy–nutrient balance (3′-Adenylic acid, adenosine, cysteine-glutathione disulfide, glutathione reduced form, Sn-Glycero-3-Phosphocholine, Trp-His, UDP-GalNAc-2Na; Figure 8B). Conversely, it showed a moderate negative correlation with metabolites implicated in lipid mobilization, structural proteolysis, and developmental resource allocation (4-Hydroxyretinoic Acid, Hyp-Ser, Ile-Ser; Figure 8B). These results suggest that a metabolic trade-off governs goose egg quality during storage.

## 4. Discussion

This study provides a comprehensive characterization of physicochemical quality changes and yolk metabolomic remodeling in Taihu goose eggs during prolonged refrigerated storage. By integrating conventional quality indices with untargeted LC-MS/MS-based metabolomics, we demonstrate that although macroscopic quality parameters remain relatively stable during early storage (Figure 2), substantial biochemical remodeling occurs progressively, with a pronounced transition emerging between 30 and 60 days of refrigeration (Figure 3 and Figure 4). These results indicate that metabolic alterations precede overt quality deterioration and may provide early insight into freshness decline in goose eggs.

### 4.1. Overall Metabolic Remodeling During Refrigerated Storage

The observed temporal patterns indicate that refrigerated storage induces stage-specific metabolic restructuring of goose egg yolk. Early storage (Day 15) was characterized by relatively conserved amino acid metabolism and central energy pathways, consistent with previous observations in chicken and duck eggs during initial cold exposure [7,14,15]. In contrast, later storage stages (Day 30–60) exhibited pronounced perturbations in lipid- and nucleotide-related metabolites (Figure 5), coinciding with significant declines in yolk color and Haugh unit (Figure 2). This temporal association supports the view that biochemical remodeling intensifies during prolonged storage and is closely linked to subsequent physicochemical quality loss.

Physicochemical measurements further suggest that goose eggs display species-specific storage characteristics. Goose eggs maintained relatively stable weight and shell integrity throughout refrigeration, and shell thinning progressed more slowly than reported for chicken eggs [14], potentially reflecting differences in shell pore architecture and ultrastructure [16]. Nevertheless, notable internal quality changes were observed. The 31.7% reduction in Haugh unit between 30 and 60 days (Figure 2A) exceeded values reported for other avian species [17], indicating increased albumen destabilization during late storage. Concurrent albumen reduction and yolk mass increase suggest intensified unidirectional moisture migration, which was more pronounced than that reported for chicken or duck eggs [18]. In parallel, a 6.7% loss of yolk pigmentation (Figure 3F) indicates accelerated carotenoid oxidation, consistent with the characteristically high unsaturated fatty acid content of goose egg yolk relative to other poultry eggs [19,20]. Recent studies have also highlighted that bioactive compounds in egg yolk, such as phospholipids, are susceptible to storage-induced degradation, which aligns with the lipid metabolic shifts observed in our study [21].

### 4.2. Purine Metabolism Disruption as a Central Metabolic Feature

Among all altered metabolic pathways, purine metabolism emerged as the most consistently and significantly perturbed during refrigerated storage (Figure 6). This observation was supported by both multivariate and univariate analyses and distinguishes goose eggs from previous poultry egg studies in which nucleotide-related alterations were reported but not identified as the dominant metabolic feature [9,22]. Notably, perturbation of purine-related metabolites became particularly evident after 30 days of storage, temporally corresponding to the onset of accelerated yolk quality deterioration.

Purine metabolites play essential roles in nucleotide turnover, redox balance, and energy-related biochemical processes. Their alteration during storage is therefore likely to reflect cumulative oxidative stress and non-enzymatic degradation within the yolk matrix rather than active metabolic regulation. For instance, the accumulation of Cysteine-glutathione disulfide observed in late storage (Figure 5F) suggests a disruption in redox homeostasis. While lipid peroxidation pathways appear broadly conserved across poultry species [23,24,25,26], the delayed onset and progression of oxidative deterioration observed in goose eggs highlight their enhanced intrinsic stability, potentially associated with higher antioxidant content and yolk structural proteins such as ovoinhibitor [15].

### 4.3. Biochemical Interpretation and Biomarker Relevance of 2-Aminopurine

A key finding of this study is the consistent and progressive depletion of 2-aminopurine across all refrigerated storage intervals (Days 15, 30, and 60 relative to Day 1) (Figure 7E). As one of only two universally altered metabolites, 2-aminopurine occupied a central position within the perturbed purine metabolic network and exhibited strong correlations with multiple yolk quality indices (Figure 7). These characteristics indicate that 2-aminopurine is a cold storage-sensitive metabolite closely associated with freshness deterioration.

From a biochemical perspective, 2-aminopurine is a critical intermediate in nucleotide salvage pathways [27]. Its progressive depletion during refrigeration may reflect cumulative oxidative degradation and altered nucleotide recycling. In addition, disruption of purine metabolism may indirectly facilitate microbial utilization of nucleotide substrates, thereby contributing to spoilage-associated biochemical changes [28,29], although this microbial link remains speculative in the absence of microbiome data in the current study. Given the untargeted and correlative nature of the present study, causal relationships cannot be established. Accordingly, 2-aminopurine should be regarded as a candidate freshness biomarker, pending further validation using targeted quantitative approaches and independent sample sets.

### 4.4. Comparison with Chicken and Duck Egg Studies

Comparative analysis suggests that goose eggs share several storage-associated features with other poultry eggs, including lipid oxidation and nucleotide alterations during prolonged refrigeration [9,22,23]. However, the timing and dominant metabolic characteristics differ among species. Whereas chicken eggs typically exhibit pronounced quality deterioration within approximately 14 days and duck eggs within 21 days [9,30], goose eggs demonstrated a delayed metabolic and physicochemical transition, occurring primarily after 30 days. Furthermore, goose eggs exhibited minimal changes in inosine monophosphate (IMP) (Figure 5B), possibly due to higher baseline levels of guanine derivatives in yolk [31], highlighting species-specific nucleotide profiles that may modulate storage resilience.

### 4.5. Limitations and Future Perspectives

Several limitations should be acknowledged. First, the untargeted metabolomic approach does not permit absolute quantification of metabolites or direct causal inference. Targeted validation of 2-aminopurine using quantitative assays and independent cohorts will therefore be essential to confirm its robustness as a freshness indicator. Second, seasonal variation, dietary factors, and microbial contributions were not explicitly examined and may influence storage-associated metabolic trajectories. Thirdly, regarding the assessment of lipid oxidation, this study primarily relied on untargeted metabolomics to screen for molecular markers rather than traditional biochemical assays such as TBARS or Peroxide Value (POV). While the absence of TBARS data limits the comparison with some classical food science literature, the metabolomic data successfully identified specific oxidation products (e.g., TMAO and amino acid derivatives) that serve as sensitive molecular signatures of yolk deterioration. Future studies integrating targeted metabolomics with multi-omics approaches, including proteomics and transcriptomics, as well as interspecies comparisons, will be valuable for distinguishing universal versus species-specific mechanisms governing egg quality during refrigerated storage.

## 5. Conclusions

This study elucidates the metabolic dynamics associated with the deterioration of goose egg yolk during storage. Metabolomic profiling identified 2-aminopurine as a novel biomarker of freshness loss, linked to disruption of purine metabolism. These findings bridge a key knowledge gap in post-harvest egg aging, directly connecting yolk metabolic alterations to macroscopic quality decline. This work provides a foundation for targeted preservation strategies and mechanism-based quality monitoring. The generalizability of these findings may be limited by the single-breed, single-origin design, and some metabolites may have evaded detection. Future research should validate the biomarker across storage conditions and egg types and explore interventions for the identified pathway to enable practical translation.

## Data Availability

The original contributions presented in this study are included in the article/Supplementary Material. Further inquiries can be directed to the corresponding authors.

## Supplementary Materials

The following supporting information can be downloaded at: https://www.mdpi.com/article/10.3390/foods15030588/s1, Supplementary File S1: Metabolites List.
